# Isolation of Secondary Metabolites From Marine Actinobacterium of Microbispora sp.T3S11 and Their Antibacterial Activities

**DOI:** 10.7759/cureus.56680

**Published:** 2024-03-22

**Authors:** Aardra B S, Vasugi Suresh, Menaka S, Pitchiah Sivaperumal

**Affiliations:** 1 Physiology, Saveetha Dental College and Hospitals, Saveetha Institute of Medical and Technical Sciences, Saveetha University, Chennai, IND; 2 Medical Physiology, Saveetha Dental College and Hospitals, Saveetha Institute of Medical and Technical Sciences, Saveetha University, Chennai, IND; 3 Prosthodontics, Saveetha Dental College and Hospitals, Saveetha Institute of Medical and Technical Sciences, Saveetha University, chennai, IND

**Keywords:** klebsiella pneumoniae, antibacterial activity, staphylococcus aureus, streptococcus mutans, agar disc assay, marine actinobacterium, microbispora sp. t3s11, secondary metabolites

## Abstract

Introduction

Marine actinobacteria are promising sources of novel bioactive compounds due to their distinct ecological niches and diverse secondary metabolite production capabilities. Among these, *Microbispora* sp. T3S11 is notable for its unique spore chain structure, which allows for both morphological and genetic identification. Despite its potential, little is understood about the secondary metabolites produced by this strain. In this study, we hope to fill this gap by extracting and analyzing the antibacterial activities of secondary metabolites from *Microbispora* sp. T3S11, which will be the first time its bioactive compound profile is investigated.

Aim

To evaluate the antibacterial activity of secondary metabolites isolated from the marine actinobacterium *Microbispora* sp. T3S11.

Materials and methods

The antibacterial assays were carried out on agar plates containing the appropriate media for each pathogen. Sterile filter paper disks were impregnated with secondary metabolites extracted from *Microbispora* sp. T3S11 and placed on the surface of agar plates inoculated with the appropriate pathogens. Similarly, disks containing tetracycline were used as a positive control. The plates were then incubated at the appropriate temperature for each pathogen, and the zones of inhibition around the disks were measured to determine the extracted metabolites' antibacterial activity.

Result

Secondary metabolites had antimicrobial activity against *Streptococcus mutans*, *Klebsiella pneumonia*, and methicillin-resistant *Staphylococcus aureus* (MRSA). The inhibition of *S. mutans* was 7.5 mm and 8.5 mm at 75 μg/mL and 100 μg/mL, respectively. *Klebsiella pneumonia* zones measured 7 mm and 7.5 mm, while MRSA zones measured 7.6 mm and 8.5 mm at the same concentrations. Tetracycline, the standard antibiotic, had larger inhibition zones: 22 mm for *S. mutans* and *Klebsiella pneumonia *and 16 mm for MRSA, indicating variable susceptibility.

Conclusion

We conclude that the secondary metabolites extracted from *Microbispora* sp. T3S11 exhibits high antibacterial activity. This could be attributed to the presence of various active compounds.

## Introduction

Chemicals known as antibiotics are those that stop other organisms from growing. These antibiotics are produced by filamentous fungi and bacteria alike, but a major source of bioactive molecules is actinomycetes, particularly the genus *Streptomyces*, where each strain is thought to produce 10-20 secondary metabolites. These metabolites have a variety of biological activities that have been reviewed, including antimicrobial, antitumor, and anti-inflammatory properties [1]. *Microbispora* is a genus of bacteria that is non-motile and Gram-positive. It belongs to the family Streptosporangiacea. They produce characteristic paired spores, which is why they are termed *Microbispora* sp. T3S11 has not yet been classified, which explains the lack of a specific name. The taxonomic ID is 1501157. They are currently characterized as high guanine (G)+cytosine (C) Gram-positive bacteria [2].

A secondary metabolite is any compound or chemical produced by an organism that is not directly involved in the growth or development of the organism. They are produced as weapons of competition against bacteria, fungi, insects, plants, and animals. Secondary metabolites play important roles in the defense and survival of organisms. They are produced in the highest amounts when an organism transitions into secondary growth from primary growth. It is mainly classified into three groups: terpenes, such as sterols, glycosides, and carotenoids; phenolics such as coumarins, lignans, tannins, flavonoids, stilbenes, and lignin; and nitrogenous compounds, such as glucosinolates and alkaloids. These groups of secondary metabolites can be easily separated by various solvent-spray systems [3].

Bacterial secondary metabolites are low molecular weight products that are vital to human health but not necessary for the growth of the producing cultures [4]. Recently, a great deal of attention has been given to marine biolife, especially rare actinomycetes such as *Microbispora*, due to their considerable biodiversity [5]. The chemical diversity of the active compounds, such as secondary metabolites, which are isolated from marine compounds, makes them a promising new source for novel antibiotics and pharmaceuticals [6]. *Microbispora* is a valuable resource for the discovery of bioactive secondary metabolites, with bispolides, linfuranone A, and microbiaeratin representing only a portion of its potential. Continued research into the diversity of *Microbispora* species is expected to yield new compounds with applications in medicine, agriculture, and industry. This review emphasizes the significance of investigating microbial biodiversity for the sustainable production of bioactive molecules. In-silico biosynthetic predictions derived from genome mining data are commonly used to identify promising *Microbispora* species in nature [7]. Secondary metabolites derived from the *Microbispora* genus have demonstrated antibacterial efficacy against various strains, including *Escherichia coli*, *Staphylococcus aureus*, *Streptococcus mutans*, *Pseudomonas aeruginosa*, and *Candida albicans*, among others [8].

The microorganisms are more difficult to get rid of when they are present as biofilms because they are resistant to antimicrobial treatments [9]. Antimicrobials are agents that kill germs or halt their growth. Antimicrobial drugs can be categorized based on the microorganisms for which they are most effective against various diseases [10]. Antibiotics have been developed and utilized to treat a variety of microbiological infections during the past century. However, it has become more and more important to find and create alternative antimicrobial medicines in recent years as a result of the advent of microbiome strains that are resistant to antimicrobial treatments [11]. The abuse and overuse of antibiotics have been implicated in the rise in the frequency of clinically documented cases of antibiotic resistance. Certain nations allow the sale of antibiotics without a prescription over the counter [12]. Multidrug-resistant bacterial strains have emerged as a result of the increasing use of antibiotics [13].

The antibacterial activity of most compounds is analyzed by the Kirby-Bauer disk diffusion method, where the pathogen is taken and inoculated into a culture medium and dispensed in a disk. The different concentrations of the experimental sample and the control sample are seeded at different points on the pathogen disk. The zones of inhibition are then visualized and measured using this method [14]. The aim of this study is to analyze the antibacterial activity of the secondary metabolites isolated from the unclassified T3S11 species of marine actinobacterium *Microbispora*.

## Materials and methods

Preparation of actinobacteria specimen

A fresh specimen of marine actinobacteria, *Microbispora* sp. T3S11, was isolated from marine seawater and identified using the spore chain morphology. After sampling, the specimen was stored in sterile test tubes at 4°C until further use.

Preparation of culture medium

The culture medium of yeast malt broth was prepared by mixing 0.8 g of glucose, 0.8 g of yeast, and 2 g of malt for every 1200 mL of broth. The marine actinobacterium *Microbispora* was inoculated into the prepared broth and allowed to grow for a week.

Isolation of secondary metabolites

**Figure 1:**
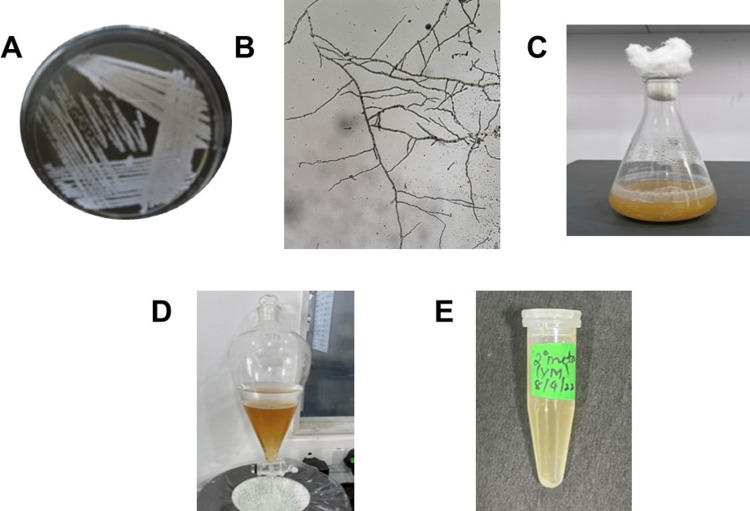
(A) Microbispora, (B) Spore Chain Morphology, (C) Mature Culture, (D) Separation of Secondary Metabolites, (E) Crude Metabolites

The mature sample of actinobacteria in the broth was centrifuged, and the supernatant and the pellet were separated. Two hundred fifty milliliters of equal volumes of the supernatant and ethyl acetate were mixed together and shaken in a separating funnel. In the separating funnel, a white layer was obtained, which contained the secondary metabolites from the actinobacterium. The white layer of secondary metabolites was separated and dried (Figure 1).

Antibacterial assay

The antibacterial properties of secondary metabolites were determined using the agar disk method. Gram-positive bacteria, such as *S. mutans* and methicillin-resistant *Staphylococcus aureus* (MRSA), as well as one Gram-negative bacteria strain, *Klebsiella pneumoniae*, were used to assess antibacterial activity. The nutrient and Müller-Hinton agar mediums were prepared and used during the experiments. Tetracycline, a standard antibiotic, was used as the positive control at a concentration of 10 μg/mL, with the blank serving as the negative control. The secondary metabolites were added to the Müller-Hinton agar at concentrations of 75 and 100 μg/mL. Petri dishes containing bacterial lawns were inoculated with 1 mL solutions of the aforementioned concentrations plus tetracycline. The plates were then incubated at 37 °C for 48 hours. The resulting inhibition zones around each paper disk were determined by measuring the diameter of the zone.

## Results

The antimicrobial activity of secondary metabolites extracted from the *Microbispora sp.* T3S11 against *S. mutans*, *K. pneumoniae*, and MRSA was investigated, revealing distinct zones of inhibition at different concentrations. The zones of inhibition of *S. mutans*, *K. pneumoniae*, and MRSA treated with secondary metabolites were compared with those treated with the standard antibiotic tetracycline.

Table 1 illustrates that the *S. mutans* inhibition zones measured 7.5 mm and 8.5 mm at concentrations of 75 μg/mL and 100 μg/mL, respectively, with a standard error of ±1 mm. For *K. pneumoniae*, inhibition zones of 7 mm and 7.5 mm were observed at the same concentrations, with a standard error of ±0.2 mm. MRSA exhibited inhibition zones of 7.6 mm and 8.5 mm at concentrations of 75 μg/mL and 100 μg/mL, respectively, with standard errors of ±1 mm and ±0.2 mm. These findings underscore the varying susceptibility of the tested bacteria to both secondary metabolites of *Microbispora sp. T3S11* and conventional antibiotics. The recorded values represent means with standard deviations where n = 3. P values were calculated using a one-tailed paired t-test, with significance denoted by *P < 0.01.

**Table 1:** Zones of inhibition of Streptococcus mutans, Klebsiella pneumoniae​​​​​​​, and MRSA MRSA: methicillin-resistant *Staphylococcus aureus*

Secondary metabolite concentration (µg/mL)	75	100	Standard-Tetracycline (10 µg/disc)
Streptococcus mutans	(7.5±1) mm	(8.5±1) mm	22 mm
Klebsiella pneumoniae	(7.0±0.2) mm	(7.5±0.2) mm	22 mm
MRSA	(7.6±1) mm	(8.5±0.2) mm	16 mm

## Discussion

According to previous research, the production of secondary metabolites is a phenotypic trait unique to certain species and is associated with widely distributed bacterial populations [15]. Actinobacteria are a diverse group of bacteria known for producing a wide range of secondary metabolites with a variety of biological functions. The production of secondary metabolites by these microorganisms is critical to their ecological function and interaction with other organisms in their environment [16]. These secondary metabolites frequently serve ecological functions, such as defense mechanisms against competing microorganisms. Furthermore, many actinobacterial secondary metabolites have found applications in medicine, agriculture, and industry due to their pharmacological and biotechnological importance. These compounds’ diverse chemical structures contribute to a wide range of activities, including antibacterial, antifungal, antiviral, and anticancer properties [17]. Secondary metabolites’ antibacterial and antimicrobial properties have the potential to be useful in the treatment of a variety of infectious diseases that are resistant to current antibiotics. This could enhance our efforts to provide alternative medical treatments, particularly in developing countries where access to healthcare is limited [18]. The primary active compound present in the extract of *Microbispora* sp. AL22 is 3,4-dihydrolactucin. This compound has been found to exhibit potent antibacterial activities as well as potent anticancer activities in various studies. The majority of the phytochemical activities of *Microbispora* species are because of the active diterpene found in *Microbispora* called 2 - alpha-hydroxy - 8(14), 15 - pimaradien - 17, 18 - dioic acid [19]. Terpenes are synthesized via two distinct biological pathways: the mevalonic acid pathway, which produces sesquiterpenes, sterols, and ubiquinones, and the methylerythritol phosphate pathway, which produces hemiterpenes, monoterpenes, and diterpenes [20]. Diterpenes, naturally occurring compounds found in plants, fungi, and marine organisms, exhibit antibacterial properties through various mechanisms. They can disrupt bacterial cell membranes, inhibit protein and nucleic acid synthesis, and induce oxidative stress, ultimately leading to bacterial cell death. Some diterpenes also act as enzyme inhibitors and may modulate immune responses. Their diverse structures enable selective activity against bacteria while minimizing toxicity to mammalian cells [21].

Recent research has shown that the marine actinobacterium *Microbispora* sp. T3S11 has significant antibacterial activity. Notably, this is the first study to document the isolation of secondary metabolites from *Microbispora* sp. T3S11. A disk diffusion assay, like the Kirby-Bauer method, can have different zones of inhibition depending on how susceptible the bacteria are to a specific antimicrobial agent. The unobstructed regions surrounding the antibiotic disks that prevent bacterial growth are known as the zones of inhibition. The zone’s size reveals how well the antibiotic works against that particular strain of bacteria [22]. The zone of inhibition for different concentrations of secondary metabolites against *S. mutans*, *K. pneumoniae*, and MRSA, along with the zone of inhibition for the standard antibiotic tetracycline against these bacteria, were analyzed [23]. For *S. mutans*, the zone increased from 7.5 mm at 75 μg/mL to 8.5 mm at 100 μg/mL. *K. pneumoniae* exhibited a similar pattern, with a rise from 7 mm to 7.5 mm at the respective concentrations. MRSA also displayed an increase from 7.6 mm to 8.5 mm, with standard errors providing a measure of result reliability (±1 mm and ±0.2 mm). Comparatively, the standard antibiotic tetracycline showcased robust inhibitory performance, with consistent zones of inhibition: 22 mm for both *S. mutans* and *K. pneumoniae*, and 16 mm for MRSA. These findings suggest a potential antibacterial effect of the secondary metabolites, albeit with varying degrees of potency across different bacterial strains. This is a very preliminary study into the antibacterial activity of the secondary metabolites extracted from *Microbispora* sp. T3S11. Further studies need to be conducted into the exact active compounds present in the species to find the source of all the phytochemical activities.

Limitations

The limitation of this study is that the T3S11 strain of *Microbispora *has not been taxonomically classified, and therefore not very much is known about the active compounds present in this particular species. The phytochemical activities of the species are also unknown. An extensive investigation needs to be conducted for the proper taxonomic classification of the T3S11 species of *Microbispora* and its phytochemical activities. The present study evaluates the antibacterial activity of II secondary metabolites of *Microbispora* sp. T3S11 by in vitro models. Subsequent research aimed at elucidating the mechanism of action, assessing efficacy, and determining toxicity in in vivo models will provide additional supporting evidence.

## Conclusions

The secondary metabolites extracted from *Microbispora* sp. T3S11, an unclassified marine actinobacterium, has significant antimicrobial activity against a variety of bacterial strains, including *S. mutans*, *K. pneumoniae*, and MRSA. The efficacy of these metabolites was determined using agar disk diffusion assays, which revealed different inhibitory zones based on concentration. The secondary metabolites had distinct antibacterial profiles when compared to the standard antibiotic, tetracycline, indicating that they could be effective antimicrobials. These promising findings highlight the need for additional research and development into the therapeutic applications of these secondary metabolites.
